# Gramine Suppresses Cervical Cancer by Targeting CDK2: Integrated Omics-Pharmacology and In Vitro Evidence

**DOI:** 10.3390/cimb48010064

**Published:** 2026-01-06

**Authors:** Zhiyan Zhou, Jin Li, Xingji Zhao, Hongxia Xu, Yu Xiao, Hongchen Wang, Ying Chen

**Affiliations:** 1 College of Basic Medical Sciences, China Three Gorges University, Yichang 443002, China; 2 College of Life Science, Yangtze University, Jingzhou 434025, China

**Keywords:** cervical cancer, gramine, network pharmacology, CDK2, ceRNA

## Abstract

Cervical cancer (CC) remains a common malignant tumor that seriously threatens women’s health globally. Gramine (GR), a natural alkaloid derived from plants such as *Arundo donax* L., exhibits anti-tumor activities, yet its mechanistic actions in CC are still unclear. Here, we integrated cell-based assays, network pharmacology, and multi-omics analysis to systematically investigate the molecular mechanisms underlying GR’s anti-CC effects. In vitro experiments showed that GR significantly inhibited proliferation and migration, induced apoptosis, and triggered G_0_/G_1_ phase cell cycle arrest in HeLa cells. Integrated multi-omics analysis identified CDK2 as a critical target of GR, with both mRNA and protein levels markedly reduced following treatment. Mechanistically, GR likely suppresses CC progression by modulating the “CYP4A22-AS1/LINC00958–hsa-miR-133b–CDK2” competitive endogenous RNA (ceRNA) axis. Immune analysis indicated positive correlations of CDK2, CYP4A22-AS1, and LINC00958 with the immune checkpoint molecule CD47. Collectively, our findings demonstrate that GR inhibits CC through a ncRNA-mediated suppression of CDK2, leading to reduced HeLa cell proliferation and migration and enhanced apoptosis. These results provide a mechanistic rationale for developing GR as a candidate agent for targeted therapy and immuno-combination strategies in CC.

## 1. Introduction

Cervical cancer (CC) ranks as the fourth leading cause of cancer-related mortality among women worldwide, surpassed only by breast, colorectal, and lung cancers. Globally, epidemiological data indicate that over 300,000 women die from CC each year [1]. In developing countries, limited access to healthcare interventions such as vaccination and screening sustains high incidence and mortality rates [2]. For decades, platinum-based doublet chemotherapy has served as the cornerstone first-line treatment for advanced CC, yet clinical outcomes remain suboptimal. Thus, exploring effective therapeutic strategies to reduce mortality is an urgent priority. Recent studies have identified numerous plant-derived bioactive compounds with potent anti-CC activity, which can markedly suppress the expression of pivotal oncogenic proteins, thereby offering promising directions for the development of novel complementary therapies [3].

Gramine (GR) is a natural indole alkaloid primarily extracted from *Arundo donax* L., though it can also be synthesized or obtained from other plants such as barley, ensuring its abundant supply [4]. This compound exhibits favorable lipophilicity, enabling efficient membrane permeability and thus facilitating its biological effects [5]. Accumulating evidence has shown that GR and its derivatives possess diverse pharmacological activities, including anti-inflammatory, antiviral, antioxidant, vasodilatory, and antitumor effects [6]. In oral squamous cell carcinoma, GR has been reported to inhibit key signaling pathways such as PI3K/Akt/mTOR and JAK/STAT3, thereby suppressing inflammatory and proliferative responses. It also modulates TGF-β signaling to inhibit tumor angiogenesis and induce apoptosis [4,7]. Furthermore, several GR analogs and conjugates have demonstrated pronounced anti-proliferative activity [8,9,10], highlighting the potential of this compound class in anti-cancer drug development. Nevertheless, the molecular mechanisms by which GR exerts effects in CC remain undefined and warrant further investigation.

Non-coding RNAs (ncRNAs) refer to a class of RNA molecules that do not encode proteins but play crucial regulatory roles in various biological processes. Based on their length, ncRNAs can be mainly divided into small non-coding RNAs (e.g., microRNAs) and long non-coding RNAs (lncRNAs), with other types such as circular RNAs (circRNAs) also included. ncRNAs can finely regulate gene expression at the transcriptional, post-transcriptional, and epigenetic levels, and are involved in tumorigenesis and cancer progression [11,12,13,14,15]. A key regulatory mechanism mediated by ncRNAs is the competitive endogenous RNA (ceRNA) network: lncRNAs can act as “molecular sponge” to adsorb microRNA, thereby relieving microRNA-mediated repression of target genes and indirectly regulating gene expression. Conversely, microRNAs (miRNAs) mainly bind to the 3′ untranslated region (3′UTR) of target mRNAs, promoting their degradation or translational inhibition, which ultimately affects tumor cell behaviors such as proliferation, apoptosis, and invasion [16,17,18]. In CC, numerous lncRNAs (e.g., HOTAIR, MALAT1) and miRNAs (e.g., miR-21, miR-34a) have been validated as oncogenes or tumor suppressors, regulating tumor progression and microenvironment remodeling through complex ceRNA networks [19,20,21,22,23,24]. In-depth analysis of dysregulated ncRNAs and their associated ceRNA circuitry in CC not only uncovers novel pathogenic mechanisms but also provides opportunities for diagnostic biomarker and therapeutic target development. Moreover, exploring the mechanisms of natural compounds like GR from a ceRNA perspective can systematically reveal their regulatory RNA networks, offering new insights into the anti-tumor mechanisms of natural medicines.

Herein, we systematically investigate the anti-CC mechanism of GR through a multi-omics integrative strategy for the first time. By integrating network pharmacology-based target prediction with bioinformatics analyses of key pathways and ncRNA networks, we identified critical targets and characterized their immune infiltration features, which were subsequently validated in CC HeLa cells. Our work elucidates a novel mechanism of GR involving core target inhibition and reveals its potential in ceRNA regulation and immune microenvironment remodeling, thereby providing a foundational rationale for developing GR as a candidate for targeted and immune-combination therapies in CC.

## 2. Materials and Methods

### 2.1. Cell Culture

The human CC HeLa cell line was acquired from the Shanghai Institute of Biochemistry and Cell Biology, Chinese Academy of Sciences. Cells were maintained in DMEM complete medium (Servicebio, Wuhan, China) supplemented with 10% fetal bovine serum (FBS, Servicebio) and incubated at 37 °C in a humidified atmosphere containing 5% CO_2_.

### 2.2. Reagents

Gramine (Catalog No.: B20777, CAS: 87-52-5) was sourced from Yuanye Bio-Technology Co., Ltd. (Shanghai, China) as an analytical standard with a purity of ≥98% (HPLC). The Cell Counting Kit-8 (CCK-8) was obtained from Sevenbio (Beijing, China). Anti-CDK2 and anti-β-actin antibodies were procured from Proteintech (Wuhan, China) and Servicebio, respectively. RT-qPCR kits (AT341, AQ602) and miRNA Reverse Transcription Kit (AT351) were purchased from TransGen Biotech (Beijing, China). The Apoptosis Assay Kit (A214) and HiScript II Q RT SuperMix for qPCR (R223) were acquired from Vazyme (Nanjing, China).

### 2.3. CCK-8 Assay

HeLa cells were seeded into 96-well plates at 5000 cells per well in 100 µL of culture medium. After 24 h of adhesion, cells were treated with various concentrations of GR (0, 70, 105, 140, and 175 µg/mL), with three replicates per group. Following 48 h of incubation, 10 µL of CCK-8 solution was added to each well, and the plates were returned to the incubator for 0.5–1 h. Absorbance was measured at 450 nm using an EnSpire^®^ luminometer (PerkinElmer, Waltham, MA, USA).

### 2.4. Wound Healing Assay

Cells were seeded in 12-well plates at a density of 5 × 10^5^ cells/mL and cultured until >90% confluent. Five uniform, straight scratches were generated per well using a sterile 10 µL pipette tip. After washing with PBS to remove detached cells, serum-free medium containing the indicated concentrations of GR was added. Wound areas were photographed at 0 and 48 h under an inverted microscope (Olympus, Tokyo, Japan) and quantified using ImageJ software (version 1.53e, NIH, Bethesda, MD, USA).

### 2.5. Flow Cytometry

For apoptosis analysis, HeLa cells were seeded in 12-well plates at 1 × 10^5^ cells/mL. After adherence, cells were treated with GR for 48 h. Cells were then collected, washed twice with pre-cooled PBS, and centrifuged at 1800 rpm for 5 min at 4 °C. The pellet was resuspended in 1× Binding Buffer, stained with Annexin V-FITC and PI according to the manufacturer’s instructions of the Annexin V-FITC/PI Apoptosis Detection Kit (Catalog No. A214-01/02, Vazyme Biotech, Nanjing, China), and incubated at room temperature in the dark. Apoptosis rates were analyzed by flow cytometry within 1 h.

For cell cycle analysis, cells were washed with PBS, fixed in pre-cooled 70% ethanol overnight at 4 °C, and then stained with PI/RNase Staining Buffer from the Cell Cycle and Apoptosis Analysis Kit (Catalog No. C1052, Beyotime Biotechnology, Shanghai, China) and incubated at 37 °C for 30 min in the dark. Cell cycle distribution was analyzed by flow cytometry within 24 h.

### 2.6. Target Collection and Identification

The GSE63514 dataset was downloaded from the GEO database, and differentially expressed genes (DEGs) were identified using GEO2R with thresholds of |log_2_ (fold change)| ≥ 2 and *p* < 0.001. CC-related targets were retrieved from DisGeNET, GeneCards, and DrugBank databases. These were merged with the DEGs from GSE63514, and duplicates were removed to generate a consolidated list of CC targets.

The SMILES identifier of GR was obtained from PubChem and submitted to the SwissTargetPrediction database to predict targets (Probability > 0). Additional potential targets were collected from TCMSP, PharmMapper, CTD, and ChEMBL databases. All targets were standardized to official gene symbols using the STRING database, integrated, and deduplicated to yield the final set of GR targets. Overlapping targets between GR and CC were identified via a Venn diagram using the online software BioLadder (https://www.bioladder.cn), accessed on 6 November 2024.

### 2.7. GO and KEGG Enrichment Analysis and Protein–Protein Interaction (PPI) Network Construction

Overlapping targets were subjected to Gene Ontology (GO) and KEGG pathway enrichment analyses using Metascape, with thresholds of minimum overlap ≥3, *p* < 0.01, and minimum enrichment factor ≥1.5. A PPI network was constructed using the STRING database and imported into Cytoscape software (v3.10.2) for topological analysis. Hub targets were identified using the CytoHubba and CytoNCA plugins.

### 2.8. Screening of Key Targets and Molecular Docking Accessed on 6 November 2024

The random forest algorithm in R was applied to the GSE63514 dataset to rank targets identified from the PPI network. The CDCP online database was used for single-cell analysis of selected genes across different cell clusters in CC datasets. Gene expression in CC was further assessed using GEPIA2 and validated with GEO datasets. Immunohistochemistry images of key genes in CC and normal tissues were retrieved from the Human Protein Atlas database, and the average optical density (AOD) was quantified using ImageJ.

The molecular structure of GR was retrieved from TCMSP in mol2 format. The 3D structure of the core target protein was downloaded from the PDB database, and water molecules and ligands were removed using PyMol 2.6. Molecular docking was performed using AutoDock Vina in AutoDock 4.2.6, and results were visualized with PyMol 2.6.

### 2.9. Prediction of the ceRNA Network for Key Genes

miRNAs targeting the key genes were predicted using StarBase, miRmap, TargetScan, and miRWalk databases. A gene-miRNA network was constructed using Cytoscape. miRNA-seq data from CC patients were downloaded from the TCGA GDC portal, and differentially expressed (DE) miRNAs were identified using the DESeq2 package in R. Experimentally supported miRNAs from the databases were intersected with DE miRNAs. Survival analysis of miRNAs was performed using the Kaplan–Meier Plotter.

DE lncRNAs were identified from TCGA transcriptome data using DESeq2. lncRNAs interacting with the selected miRNAs were predicted using StarBase, LncBook, and NPInter. Experimentally supported lncRNAs were intersected with DE lncRNAs, and their prognostic value was assessed via survival analysis. Pearson correlation analysis was used to evaluate relationships between key genes and relevant non-coding RNAs. The final mRNA-miRNA-lncRNA network was visualized using Cytoscape.

### 2.10. RT-qPCR

HeLa cells were seeded in 24-well plates (1 × 10^5^ cells/well), treated after overnight culture, and total RNA was extracted using Trizol reagent. cDNA was synthesized using specific reverse transcription kits for mRNA, miRNA, and lncRNA. The qPCR reaction mixture was prepared with cDNA, SYBR Green master mix, specific primers (sequences in Table 1), and nuclease-free water. The thermal cycling conditions were: 94 °C for 30 s (1 cycle); 40 cycles of 94 °C for 5 s and 60 °C for 30 s; followed by a dissociation stage. Relative gene expression was calculated using the 2^−ΔΔCt^ method, and results were plotted using GraphPad Prism 9.

### 2.11. Western Blotting

Cells were seeded in 6-well plates at 1 × 10^6^ cells/mL, treated after adherence, and total protein was extracted after 48 h. Protein concentration was determined using the BCA method. Samples were denatured with Loading Buffer at 100 °C for 10 min, separated by SDS-PAGE, and transferred to PVDF membranes. Membranes were blocked with 5% skim milk for 1 h at room temperature, incubated with primary antibodies at 4 °C overnight, washed with PBST, and incubated with HRP-conjugated secondary antibodies for 1 h at room temperature. After washing, bands were detected using ECL substrate, and images were analyzed with ImageJ.

### 2.12. Immune Infiltration Analysis

Samples were divided into high- and low-expression groups based on the mean expression of target genes. Immune infiltration analysis was conducted using the CIBERSORT algorithm on TCGA data, and correlations between genes and immune cells were visualized. The TIMER3 database was used for additional immune infiltration analysis in CC. Correlation between key genes and 25 immune checkpoints was analyzed using TCGA data.

### 2.13. Statistical Analysis

Data were analyzed using GraphPad Prism 9.5.0 and presented as mean ± standard deviation ($\bar{x}\;\;\bar{x}\pm s$). Comparisons between groups were performed using Student’s *t*-test. A *p* value < 0.05 was considered statistically significant.

## 3. Results

### 3.1. GR Inhibits Proliferation, Migration, and Induces Apoptosis and Cell Cycle Arrest in HeLa Cells

**Figure 1:**
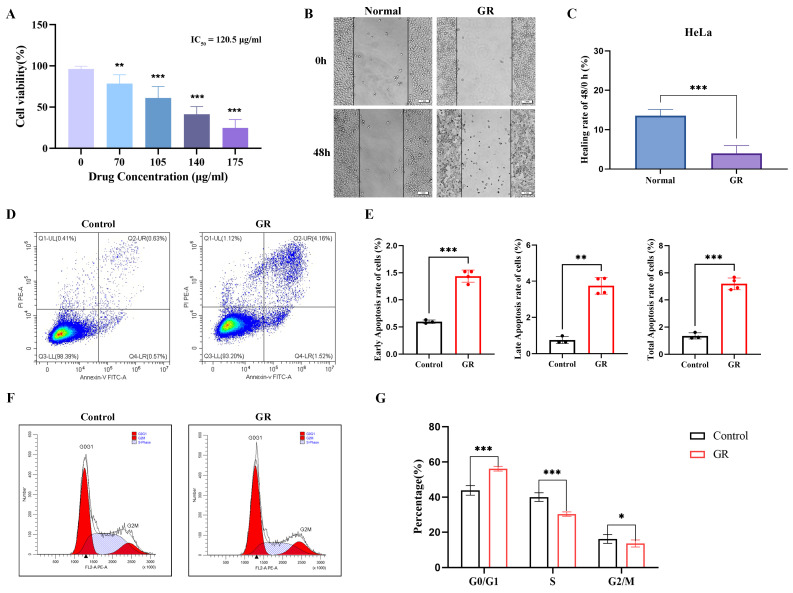
Effects of GR on HeLa cells. (**A**) Viability of HeLa cells treated with GR for 48 h, assessed by CCK-8 assay. (**B**,**C**) Representative images and quantitative analysis of cell migration evaluated by scratch assay. (**D**,**E**) Apoptosis analysis by flow cytometry with quantitative analysis. The color gradient in the dot plots (**D**) represents cell density, with brighter colors (e.g., green, yellow, red) indicating higher cell counts and darker blue indicating lower cell counts in the corresponding region. (**F**,**G**) Cell cycle distribution analyzed by flow cytometry and corresponding quantification. The triangle marker (in F) indicates the G_0_/G_1_ phase peak of the cell cycle. * *p* < 0.05, ** *p* < 0.01, *** *p* < 0.001.

Treatment of HeLa cells with increasing concentrations of GR (0–175 μg/mL) for 48 h resulted in a dose-dependent suppression of cell viability, as determined by CCK-8 assay, with a half-maximal inhibitory concentration (IC_50_) of 120 μg/mL (Figure 1A). Accordingly, a concentration of 120 μg/mL was selected for subsequent migration assays. Compared with the control group, the GR-treated cells exhibited blurred boundaries and a rounded, shrunken morphology, alongside a 9.53%reduction in migration rate (Figure S1 and Figure 1B,C). Flow cytometric analysis further revealed that GR promoted apoptosis, as evidenced by increased proportions of early-apoptotic, late-apoptotic, and total apoptotic cells (Figure 1D,E). Cell cycle profiling indicated that GR reduced the percentage of cells in S phase and G_2_/M phase by approximately 9.76% and 2.57%, respectively, while markedly elevating the G_0_/G_1_ population by about 12.32% (Figure 1F,G). Taken together, these data demonstrated that GR effectively suppressed the proliferation and migration, and concurrently triggered apoptosis and G_0_/G_1_ phase cell cycle arrest in HeLa cells.

### 3.2. Identification of GR-Associated Targets and Enrichment Analysis

**Figure 2:**
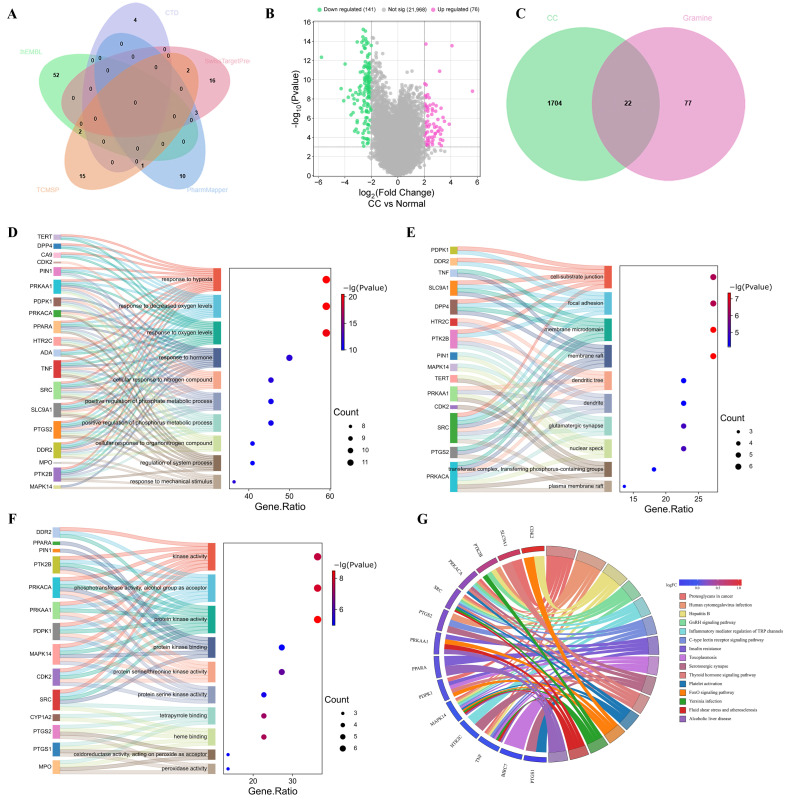
Identification of GR-Associated Targets and Enrichment Analysis. (**A**) Collection of GR-associated targets from multiple databases, represented as a Venn diagram. Each database is color-coded: ChEMBL (light green), CTD (pale purple), SwissTargetPrediction (light pink), PharmMapper (light blue), and TCMSP (light orange). The common core region is shown in light brown, with numerical labels indicating the count of unique and shared targets. (**B**) Volcano plot of differential expression analysis in the GSE63514 dataset. (**C**) Venn diagram showing the overlap between CC and GR targets. (**D**) GO-Biological Process (BP) enrichment. (**E**) GO-Cellular Component (CC) enrichment. (**F**) GO-Molecular Function (MF) enrichment. (**G**) KEGG pathway enrichment.

To elucidate the molecular mechanisms of GR in the treatment of CC, we first collected its potential targets from several databases, including TCMSP (20 targets), PharmMapper (14), SwissTargetPrediction (21), CTD (4), and ChEMBL (55). After removing duplicates, 99 unique targets were retained (Figure 2A). For CC-related targets, 72, 1517, and 28 genes were retrieved from DisGeNET, GeneCards, and Drugbank, respectively. Meanwhile, differential gene expression analysis of the GSE63514 dataset using the GEO2R tool yielded 217 differentially expressed genes (Figure 2B). After merging and deduplicating all CC-related entries, 1726 targets were acquired. By intersecting the GR and CC targets, 22 common genes were ultimately identified as potential therapeutic targets of GR against CC (Figure 2C).

Subsequently, functional enrichment analyses were performed on these 22 targets via the Metascape platform. Gene Ontology (GO) analysis revealed that, in biological processes (BP), targets were predominantly involved in the response to oxygen levels, hypoxia, and decreased oxygen levels (Figure 2D–F). For cellular components (CC), targets were mainly localized to membrane raft, membrane microdomain, and focal adhesion. Molecular functions (MF) terms were observed for protein kinase activity, phosphotransferase activity with alcohol as acceptor, and kinase activity. KEGG pathway analysis indicated significant involvement in proteoglycans in cancer, cytomegalovirus infection, and human papillomavirus infection (Figure 2G). In summary, these findings suggest that GR may modulate kinase-driven signaling within specific membrane domains, thereby facilitating cellular adaptation to hypoxia, and influencing tumorigenesis as well as virus related pathogenic mechanisms in CC.

### 3.3. CDK2 Identified as a Key Target of GR’s Anti-CC Activity

**Figure 3:**
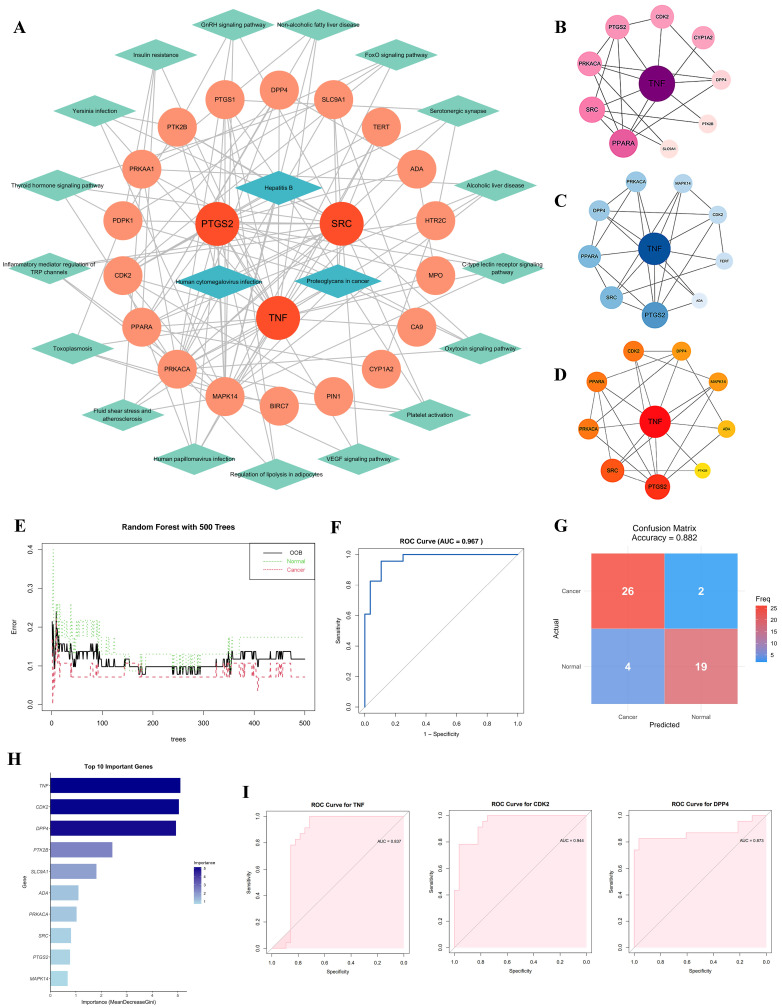
Identification of Key Targets. (**A**) Network diagram of targets and pathways for GR against CC. Circular nodes represent targets, square nodes denote related pathways. Genes are ordered by DC (Degree Centrality) values. (**B**–**D**) Top 10 targets ranked by Betweenness Centrality (**B**), Closeness Centrality (**C**), and Degree Centrality (**D**). (**E**) Random forest error curve of the optimal decision tree: the black line represents out-of-bag (OOB) error; the green and red lines show the prediction error for the cancer and normal groups, respectively. The plot illustrates error trends across the 500-tree model (x-axis: tree number, y-axis: error). (**F**) ROC curve of the random forest model, evaluating its overall classification performance (AUC = 0.987). (**G**) Confusion matrix of the predictive model. (**H**) The top 10 important genes identified. (**I**) Gene-specific ROC curves for TNF, CDK2, and DPP4, illustrating the independent diagnostic value of each gene; pink shading indicates the AUC range.

Firstly, a protein–protein interaction (PPI) network of GR’s anti-CC targets was constructed using the STRING database (Figure 3A). This network was imported into Cytoscape, and the CytoHubba plugin was employed to calculate and screen the top 10 targets based on three centrality metrics—Betweenness, Closeness, and Degree (Figure 3B–D). The union of the top-10 nodes from each metric yielded twelve core targets: TNF, PPARA, SRC, PRKACA, PTGS2, CDK2, DPP4, PTK2B, SLC9A1, MAPK14, TERT, and ADA (Table S2). These genes were further evaluated using a random forest algorithm, with feature importance ranked by the Mean Decrease in Gini index. TNF, CDK2, and DPP4 emerged as the top three candidates (Figure 3H). Receiver-operating characteristic (ROC) curve analysis demonstrated area-under-the-curve (AUC) values of 0.837, 0.944, and 0.873 for TNF, CDK2, and DPP4, respectively (Figure 3I).

**Figure 4:**
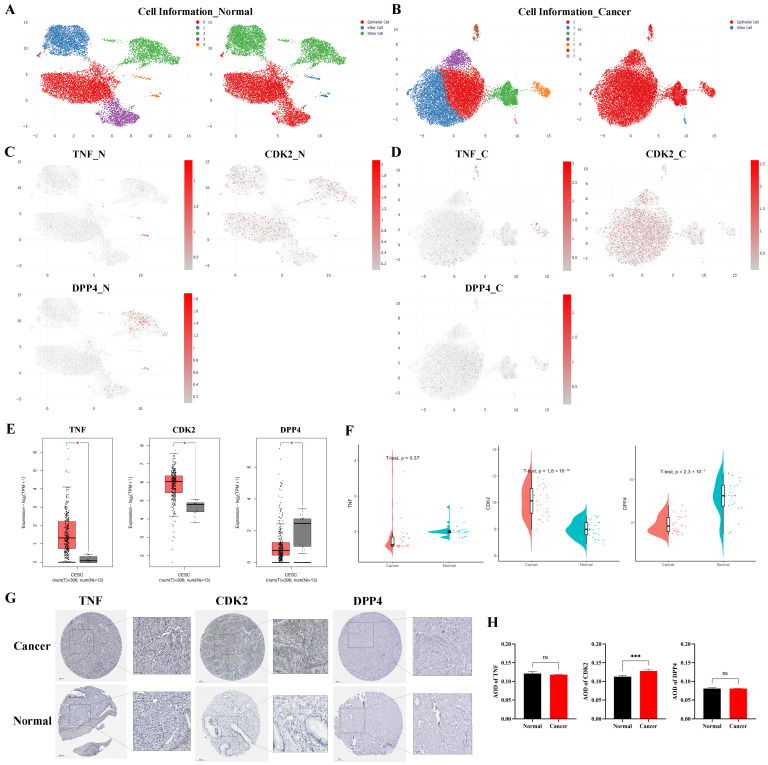
Expression profiles of the key targets. (**A**,**B**) Cell type classification in adjacent normal (**A**) and CC tissue (**B**). (**C**,**D**) Expression of TNF, CDK2, and DPP4 across cell types in adjacent normal (**C**) and CC (**D**) samples. (**E**,**F**) Differential mRNA expression in CC versus normal tissues from the GEPIA2 database ((**E**), where * denotes *p* < 0.05) and GEO dataset GSE63514 (**F**). (**G**) Representative IHC images of protein expression from the HPA database. (**H**) Quantitative analysis of IHC results based on average optical density (AOD). *** *p* < 0.001; ns, not significant (*p* ≥ 0.05).

We subsequently conducted a detailed expression analysis of these three core targets. Analysis of the single-cell RNA sequencing dataset SCDS0000550 (from the CDCP platform) revealed that, compared with normal cells, the mRNA expression levels of CDK2 and TNF were up-regulated in CC, whereas DPP4 was down-regulated (Figure 4A–D, Table S3). In epithelial cells, all three transcripts displayed an upward trend (Table S3). Analysis of bulk transcriptomic data from the GEPIA 2 database also indicated elevated mRNA levels of TNF and CDK2, alongside reduced DPP4 expression in CC tissues (Figure 4E). Differential analysis of the GSE63514 dataset further confirmed this pattern, showing upregulation of CDK2, downregulation of DPP4, and no significant change in TNF expression (Figure 4F). Moreover, immunohistochemistry data from the HPA database indicated higher CDK2 protein abundance in CC tissues, while TNF and DPP4 levels remained unchanged (Figure 4G,H).

**Figure 5:**
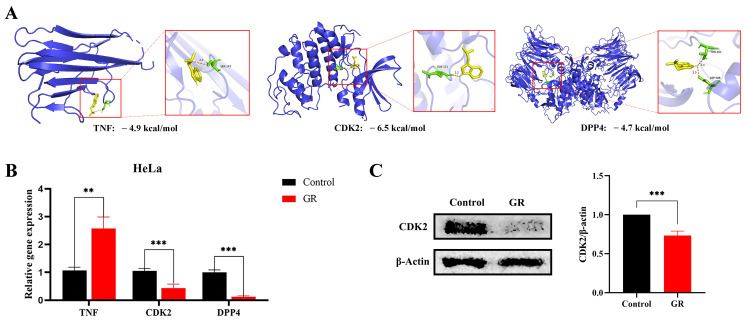
Molecular Docking and Expression Validation of Key Targets. (**A**) Molecular docking of TNF, CDK2, and DPP4 with GR. Colors indicate: purple (protein), yellow (GR), and green (key binding residues); hydrogen bonds are shown in orange. (**B**) RT-qPCR detection of TNF, CDK2, and DPP4 mRNA levels after GR treatment. (**C**) Western blotting analysis of CDK2 protein expression following GR exposure. ** *p* < 0.01, *** *p* < 0.001.

To investigate the direct targeting potential of GR, molecular docking was performed with TNF, CDK2, and DPP4 (Figure 5A). Among them, only CDK2 displayed a favorable binding energy below −5 kcal/mol (–6.5 kcal/mol), indicating a stable interaction, whereas the values for TNF (−4.9 kcal/mol) and DPP4 (−4.7 kcal/mol) were less favorable. This computational prediction was consistent with subsequent experimental results. In HeLa cells treated with GR (120 μg/mL, 48 h), RT-qPCR analysis showed a significant increase in TNF mRNA but a marked reduction in CDK2 and DPP4 transcripts (Figure 5B). Notably, the downregulation of CDK2 was further confirmed at the protein level by Western blotting (Figure 5C). Taken together, CDK2 was selected as the principal target for further mechanistic investigation.

### 3.4. Construction and Validation of a ceRNA Regulatory Network Upstream of the Core Target CDK2

**Figure 6:**
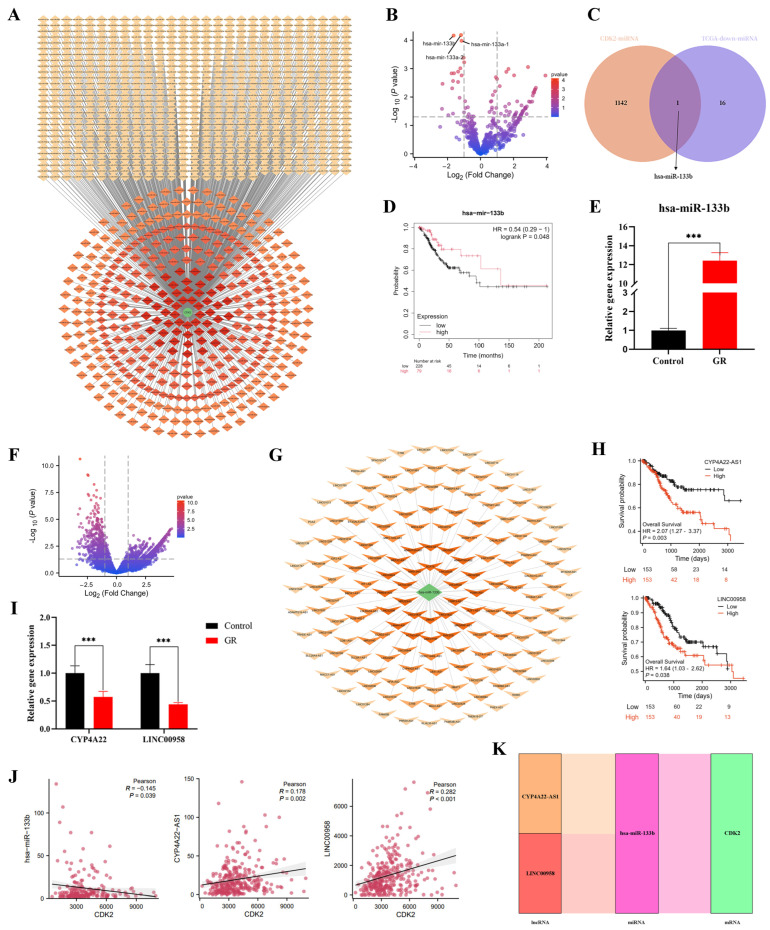
Construction and validation of the CDK2-targeting ceRNA network. (**A**) miRNA-CDK2 interaction network. (**B**) Volcano plot of differentially expressed miRNAs in the TCGA-CESC dataset. The x- and y-axes show log_2_ (Fold Change) and −log_10_ (*p*-value), respectively; the dashed lines indicate thresholds (|log_2_FC| > 1, −log_10_(p) > 1.3), solid line marks the baseline (log_2_FC = 0), and red dots represent significant hits. (**C**) Intersection between CDK2-targeting miRNAs and differentially expressed miRNAs in TCGA-CESC. Numbers indicate unique/overlapping molecule counts. (**D**) Survival curve of hsa-miR-133b. (**E**) RT-qPCR quantification of hsa-miR-133b levels following GR exposure. (**F**) Volcano plot of differentially expressed lncRNAs in the TCGA-CESC. The axes and annotations follow the same scheme as in panel B. (**G**) Network diagram of lncRNAs targeting hsa-miR-133b, sorted by *p* value. (**H**) Survival analysis of CYP4A22-AS1 and LINC00958. (**I**) Expression of lncRNAs in HeLa cells after GR treatment. (**J**) Correlation scatter plots among CDK2, hsa-miR-133b, CYP4A22-AS1, and LINC00958. Axes show expression levels (TPM/FPKM), red dots represent individual samples, the solid line is the linear regression fit, the dashed band indicates its 95% confidence interval, and in-plot values give the correlation coefficient and *p*-value. (**K**) Sankey diagram of the ceRNA regulatory axis. *** *p* < 0.001.

Non-coding RNAs can exert pivotal functions in cancer progression through competing endogenous RNA (ceRNA) mechanisms, and drug-induced modulation of cancer cells may also involve ceRNA pathways. To determine whether the core target CDK2 is regulated by a ceRNA network, we first predicted and screened miRNAs potentially target CDK2. By integrating prediction results from four databases, 1143 non-redundant miRNAs were obtained (Figure 6A). Differential expression analysis of these miRNAs in CC identified seven up-regulated miRNAs and one down-regulated miRNA (hsa-miR-133b) (Figure 6B,C; Table S4). Kaplan–Meier survival analysis demonstrated that high expression of hsa-miR-133b was significantly associated with favorable patient prognosis (Figure 6D). HeLa cells treated with 120 µg/mL GR for 48 h showed a marked increase in hsa-miR-133b levels compared with controls (*p* < 0.001) as measured by RT-qPCR (Figure 6E).

Subsequently, upstream lncRNAs predicted to bind hsa-miR-133b were identified and intersected with lncRNAs significantly up-regulated in the TCGA CC dataset, yielding 62 candidate lncRNAs (Figure 6F). After sorting by *p* value of differential expression, a regulatory network of lncRNAs targeting hsa-miR-133b was constructed (Figure 6G). Survival analysis revealed that seven lncRNAs (CYP4A22-AS1, LINC00958, LINC00885, RCCD1-AS1, LINC01206, LINC00944, LINC01010) were significantly correlated with patient outcomes (Figure S2), among which high expression of CYP4A22-AS1 and LINC00958 predicted poorer prognosis (Figure 6H). Experimental validation showed that 48 h GR treatment significantly down-regulated the expression of both CYP4A22-AS1 and LINC00958 in HeLa cells (*p* < 0.001) (Figure 6I). Correlation analysis demonstrated a negative relationship between hsa-miR-133b and CDK2, whereas CYP4A22-AS1 and LINC00958 were positively correlated with CDK2 (Figure 6J). Finally, key lncRNAs and genes (e.g., CDK2) were integrated into a Sankey diagram illustrating the ceRNA regulatory network (Figure 6K). Collectively, these results suggest that GR suppresses the malignant progression of HeLa cells by modulating the CYP4A22 AS1/LINC00958–hsa-miR-133b–CDK2 axis.

### 3.5. Immune Regulatory Profiles of CDK2, CYP4A22-AS1, and LINC00958 in CC

**Figure 7:**
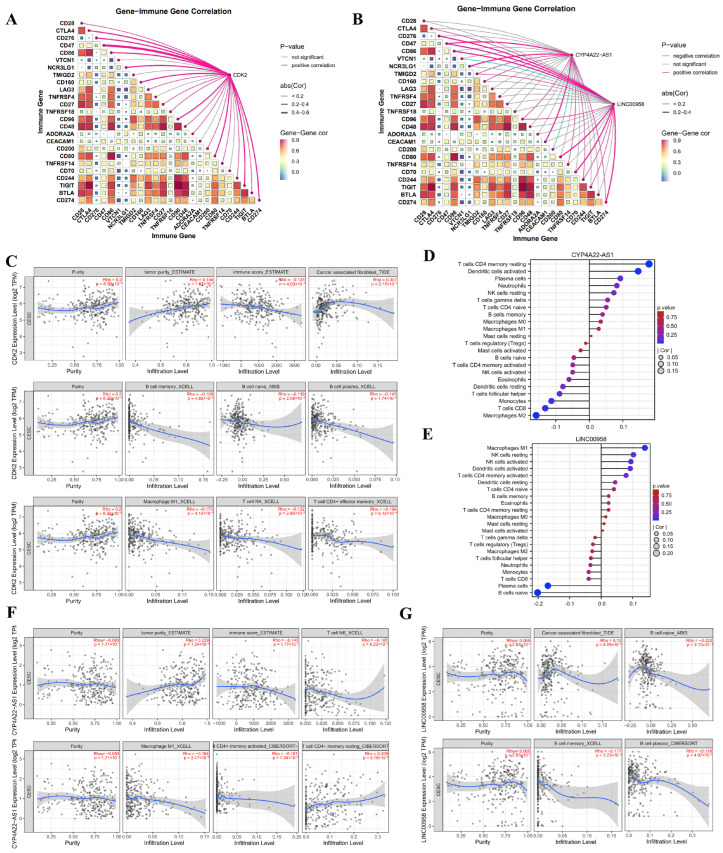
Immune infiltration analyses of CDK2, CYP4A22-AS1 and LINC00958 in CC. (**A**) Correlation between CDK2 expression and immune checkpoint molecules. (**B**) Correlation of CYP4A22-AS1 and LINC00958 expression with immune checkpoint molecules. (**C**) Comprehensive immune infiltration profile of CDK2. (**D**) Lollipop plot depicting the relationship between CYP4A22-AS1 and immune cells. (**E**) Lollipop plot illustrating the association between LINC00958 and immune-cell subsets. (**F**,**G**) Analysis of immune cell infiltration correlated with (**F**) CYP4A22-AS1 and (**G**) LINC00958, respectively, using the TIMER3 database. In panels (**C**,**F**,**G**): black dots represent individual samples; the blue solid line is the fitted regression curve; the gray shaded area indicates its 95% confidence interval.

To elucidate the immunomodulatory roles of GR-associated factors, we performed immune infiltration analyses on CDK2, CYP4A22-AS1, and LINC00958. We first examined CDK2 and observed its broad positive correlations with multiple immune checkpoint molecules, particularly CD47, CD276, and NCR3LG1 (Figure 7A; Table S5). Further assessment showed CDK2 was positively associated with tumor purity and cancer-associated fibroblast (CAF) infiltration, but negatively with immune score and NK cells, memory B cells, naïve B cells, and plasma cells (Figure 7C). These findings suggest that CDK2 may contribute to CC progression by fostering an immunosuppressive microenvironment, underscoring its potential as an immunotherapeutic target. Similarly, both CYP4A22-AS1 and LINC00958 exhibited a significant positive correlation with the immune checkpoint CD47, stronger than with other checkpoints (Figure 7B; Table S6). Integrated multi-algorithm immune profiling demonstrated that CYP4A22-AS1 was positively linked to tumor purity and resting CD4^+^ memory T cells, and negatively to immune score and NK cells. LINC00958, however, showed a positive association with CAFs but negatively with B cell lineages (Figure 7D–G). This pattern indicates both lncRNAs favor an immunosuppressive state.

Strikingly, CDK2 shared a highly similar immunoregulatory signature with both CYP4A22-AS1 and LINC00958. The concerted association of all three molecules with an immunosuppressive microenvironment suggested they may function cooperatively to facilitate immune evasion.

## 4. Discussion

CC remains a prevalent malignancy of the female reproductive system, with limited treatment options for advanced-stage patients [2,25]. GR, a natural indole alkaloid, has previously been reported to possess certain antitumor activities, yet its specific function in CC remains poorly characterized [26]. Here, we demonstrated that GR exerts potent anti-tumor effects in HeLa cells, inhibiting proliferation and migration, while inducing apoptosis and G_0_/G_1_ phase cell cycle arrest (Figure 1F,G). These observations are consistent with previously documented antitumor activities of GR in other cancer models, such as oral squamous cell carcinoma, supporting its potential as a broad-spectrum anti-cancer agent [14].

To systematically explore the molecular targets of GR, we integrated network pharmacology, PPI network analysis, Random Forest machine learning algorithms, single-cell transcriptome analysis, ROC curve evaluation, and differential gene validation, complemented by molecular docking simulations. This comprehensive approach identified CDK2 as a pivotal target. Experimentally, GR treatment markedly reduced both mRNA and protein expression levels of CDK2 in HeLa cells, establishing CDK2 as a central mediator of GR’s anti-cancer activity. This finding is consistent with previous reports that CDK2 is frequently overexpressed in CC, where it complexes with cyclin E1 to promote p-Rb phosphorylation and E2F1 release, thereby driving cell cycle progression and tumor proliferation [27,28].

MicroRNAs (miRNAs) constitute a class of crucial gene expression regulators that play key roles in tumor initiation and progression [29,30,31]. In the present study, we identified hsa-miR-133b as a potential target miRNA of CDK2, which is downregulated in CC tissues and associated with improved patient survival. In vitro experiments further confirmed that GR treatment significantly upregulated hsa-miR-133b expression in HeLa cells, suggesting that GR may indirectly inhibit CDK2 by enhancing hsa-miR-133b expression. While one study suggested an oncogenic role for hsa-miR-133b in advanced CC [32], the prevailing evidence from other reports [33,34] and its documented downregulation in various other malignancies, including prostate cancer, oral squamous cell carcinoma, and esophageal cancer [35,36,37,38], supports its function as a tumor suppressor.

Based on the ceRNA regulatory mechanism, we identified CYP4A22-AS1 and LINC00958 as lncRNAs potentially acting as molecular sponges for hsa-miR-133b. Experimental results indicated that GR treatment significantly suppressed the expression of both CYP4A22-AS1 and LINC00958 in HeLa cells. Correlation analysis revealed that CDK2 expression is negatively correlated with hsa miR 133b but positively correlated with CYP4A22 AS1 and LINC00958, suggesting that GR may relieve the “molecular sponge” sequestration of hsa miR 133b by CYP4A22 AS1 or LINC00958, thereby enhancing hsa miR 133b mediated repression of CDK2 and forming a complete “lncRNA–miRNA–mRNA” regulatory axis. Both CYP4A22-AS1 and LINC00958 have been characterized as oncogenic lncRNAs. CYP4A22-AS1 is highly expressed in lung adenocarcinoma, gastric cancer, and colorectal cancer, associated with poor patient prognosis, and promotes the proliferation and metastasis of lung adenocarcinoma [39,40,41]. Similarly, LINC00958 is upregulated in CC and facilitates drug resistance, lymph angiogenesis, proliferation, and metastasis by regulating multiple downstream target genes [42]. However, functional interactions between these two lncRNAs and hsa-miR-133b have not yet been reported.

The immune landscape associated with CDK2, CYP4A22-AS1, and LINC00958 reveals a convergent immunosuppressive role in CC. CDK2 expression is linked to higher tumor purity and CAF infiltration, but lower immune score and reduced levels of antitumor immune cells, including B cells and M1 macrophages. CAFs promote lymphatic metastasis [43] and impair B cell function via MIF-CD44 [44]; B cells (CD19^+^), in contrast, correlate with better prognosis [45]. Similarly, CYP4A22-AS1 shows a positive correlation with resting CD4^+^ memory T cells (a known risk factor) and a negative correlation with activated CD4^+^ memory T cells and M1 macrophages, whereas LINC00958 is positively correlated with CAFs and negatively with B cells. Importantly, M1 macrophages can shift to a pro-tumor M2 state [46], a process modulated by STAT signaling in CC [47].

Notably, CDK2, CYP4A22-AS1, and LINC00958 all demonstrated significant positive correlations with the immune checkpoint molecule CD47. CDK2 was also correlated with CD276 and NCR3LG1. CD47 binds SIRPα to deliver a “don’t eat me” signal that inhibits phagocytosis [48], while CD276 and NCR3LG1 belong to the B7 family, and their overexpression indicates poor prognosis [49]. Blocking the CD47–SIRPα axis can enhance phagocytosis and T cell activity, thereby delaying tumor progression [50].

Emerging evidence suggests that inhibiting CDK2 can potentiate anti-tumor immunity and synergize with immunotherapy [51,52,53]. In contrast, the immunoregulatory functions of CYP4A22-AS1 and LINC00958 remain less explored, with only one recent study indicating that exosomal LINC00958 induces M2 macrophage polarization to maintain stemness in ovarian cancer [54].

In summary, our findings indicate that CDK2, CYP4A22-AS1, and LINC00958 collectively drive CC progression by fostering an immunosuppressive microenvironment, as evidenced by their shared positive correlations with checkpoints like CD47 and pro-tumor immune cells, and negative correlations with anti-tumor immune cells. By downregulating CDK2 and these two lncRNAs, GR may simultaneously inhibit tumor proliferation and reverse the immunosuppressive state, providing a theoretical rationale for exploring its combination with immune checkpoint inhibitors in the treatment of CC.

## 5. Conclusions

**Figure 8:**
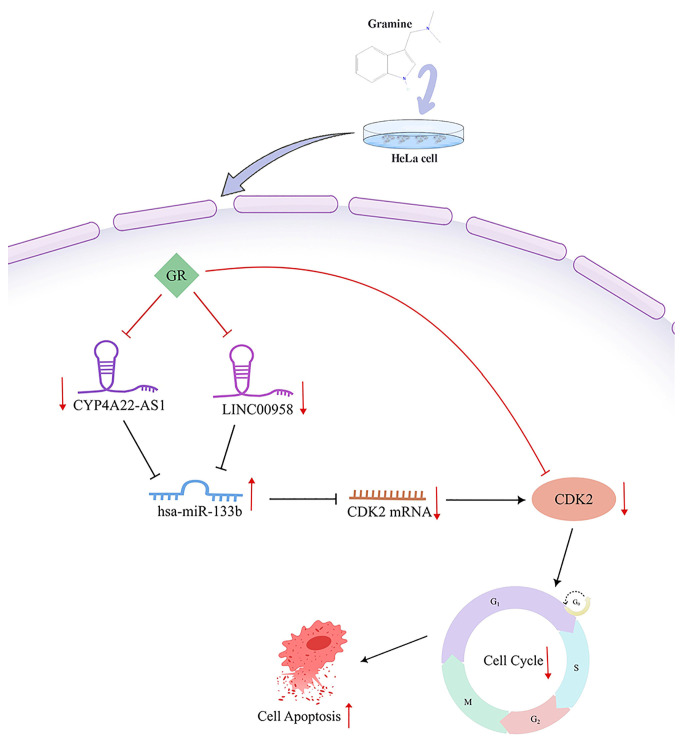
Mechanistic insight into GR-mediated regulation of CC. Colors represent: green (drug GR), purple/pink (lncRNAs), blue (miRNA hsa-miR-133b), orange (CDK2 mRNA/protein). Arrows indicate: red (up-/downregulation), flat-head (inhibition), black (promotion).

This study establishes that GR exerts anti-tumor effects in CC HeLa cells by targeting CDK2, potentially via the novel CYP4A22-AS1/LINC00958–hsa-miR-133b–CDK2 regulatory axis. The involved molecules further exhibit distinct immunomodulatory properties, suggesting a potential role in reshaping the tumor immune microenvironment (Figure 8). However, several limitations should be acknowledged. The conclusions are drawn primarily from experiments in a single cell line, and broader validation in additional models is needed. The proposed ceRNA network also requires direct experimental verification—such as by luciferase reporter and RNA immunoprecipitation (RIP) assays—and the immunomodulatory effects of GR warrant further functional investigation. Future research should focus on validating this mechanistic model and investigating GR’s synergy with existing immunotherapies. Despite these limitations, our work pinpoints a specific molecular target and a previously unreported ceRNA network for GR, providing a mechanistic basis for its development as a therapeutic candidate, particularly in combination with immune-based strategies for CC.

## Figures and Tables

**Table 1:** Sequence of mRNA, miRNA, and lncRNA Primers for qRT-PCR.

Gene	Forward 5′-3′	Reverse 5′-3′
TNF	CCTCTCTCTAATCAGCCCTCTG	GAGGACCTGGGAGTAGATGAG
CDK2	CCAGGAGTTACTTCTATGCCTGA	TTCATCCAGGGGAGGTACAAC
DPP4	GGGTCACATGGTCACCAGTG	TCTGTGTCGTTAAATTGGGCAT
CYP4A22-AS1	GTGCGTCAGAGCTAGCAAG	AGCATGGTCAGGTTGGTCAG
LINC00958	CTACACAGACGCCAGGTAGC	GGCTGGAGCCCATCCATTAG
U6	CTCGCTTCGGCAGCACA	AACGCTTCACGAATTTGCGT
hsa-mir-133b	TTGGTCCCCTTCAACCAGC	GCGAGCACAGAATTAATACGAC

## Data Availability

The original contributions presented in this study are included in the article and Supplementary Materials. Further inquiries can be directed to the corresponding authors.

## Supplementary Materials

The following supporting information can be downloaded at: https://www.mdpi.com/article/10.3390/cimb48010064/s1.

## Abbreviations

The following abbreviations are used in this manuscript:

CC	Cervical cancer
GR	Gramine
GO	Gene Ontology
KEGG	Kyoto Encyclopedia of Genes and Genomes
PPI	Protein−protein interaction
CDCP	Cell-omics Data Coordinate Platform
HPA	Human Protein Atlas
AOD	Average optical density
PDB	Protein Data Bank
CDK2	Cyclin-dependent kinase 2
TNF	Tumor necrosis factor
DPP4	Dipeptidyl peptidase 4
