# Supplementary material for: Gramine Suppresses Cervical Cancer by Targeting CDK2: Integrated Omics-Pharmacology and In Vitro Evidence
Source: Curr Issues Mol Biol. 2026 Jan 6;48(1):64. doi: 10.3390/cimb48010064 (PMC12840243; doi:10.3390/cimb48010064)
Supplement: Supplementary file 1 [file cimb-48-00064-s001.zip › cimb-4060757-supplementary.pdf]

Supplementary Table

Table S1. The 12 targets screened by PPI and their scoring points

Gene Symbol	Full Name	Score of Betweenness	Score of Closeness	Score of Degree
TNF	Tumor necrosis factor	123.300	0.731	13
PPARA	Peroxisome proliferator-activated receptor alpha	71.867	0.576	6
SRC	Proto-oncogene tyrosine-protein kinase Src	55.700	0.576	8
PRKACA	cAMP-dependent protein kinase catalytic subunit alpha	47.033	0.543	6
PTGS2	Prostaglandin G/H synthase 2	43.900	0.633	9
CDK2	Cyclin-dependent kinase 2	39.733	0.500	6
DPP4	Dipeptidyl peptidase 4	12.167	0.543	5
PTK2B	Protein-tyrosine kinase 2-beta	5.767	0.475	3
SLC9A1	Sodium/hydrogen exchanger 1	5.467	0.432	3
MAPK14	Mitogen-activated protein kinase 14	5.133	0.513	5
TERT	Telomerase reverse transcriptase	2.300	0.487	3
ADA	Adenosine deaminase	1.400	0.463	4

Table S2. Expression differences of 10 genes in single-cell analysis

	Normal		Cancer		
gene	nCells	percent	nCells	percent	logFC
DPP4	177	1.55	113	0.79	-0.292704607
MAPK14	2680	23.46	2677	18.83	-0.095477688
PRKACA	1531	13.40	1984	13.95	0.017469409
PTGS2	873	7.64	1548	10.89	0.153934521
SRC	705	6.17	1827	12.85	0.318617964
TNF	48	0.42	164	1.15	0.43744855
SLC9A1	543	4.75	2304	16.2	0.532821405
CDK2	472	4.13	2047	14.4	0.54241244
PTK2B	376	3.29	2088	14.68	0.649530158
ADA	164	1.44	2797	19.67	1.135441868

Table S3. Differential expression of 10 genes in epithelial cells

	Normal		Cancer		
gene	nExpress	pctExpress	nExpress	pctExpress	logFC
MAPK14	2000	30.42	2677	18.88	-0.20716
PRKACA	796	12.11	1984	13.99	0.062674
SRC	636	9.67	1827	12.88	0.124489
PTGS2	275	4.18	1548	10.92	0.417046
DPP4	17	0.26	113	0.80	0.488117
SLC9A1	324	4.93	2304	16.25	0.518006
CDK2	240	3.65	2047	14.44	0.597274
ADA	94	1.43	2796	19.72	1.139571
TNF	5	0.08	164	1.16	1.161368
PTK2B	52	0.79	2088	14.72	1.270281

Table S4. Results of differential analysis of cervical cancer TCGA-miRNA using the DESeq2 package

DE-miRNA	p-value	Group	DE-miRNA	p-value	Group
hsa-miR-1-1	0.000413	down	hsa-miR-944	0.000882	up
hsa-miR-129-1	0.000976	down	hsa-miR-31	0.001031	up
hsa-miR-129-2	0.001734	down	hsa-miR-203b	0.001302	up
hsa-miR-133a-1	0.000105	down	hsa-miR-96	0.001727	up
hsa-miR-133a-2	6.59E-05	down	hsa-miR-4652	0.001752	up
hsa-miR-133b	6.88E-05	down	hsa-miR-429	0.004142	up
hsa-miR-204	0.001282	down	hsa-miR-548j	0.004737	up
hsa-miR-3199-1	0.002732	down	hsa-miR-4664	0.005411	up
hsa-miR-3622a	0.003436	down	hsa-miR-4638	0.005491	up
hsa-miR-372	0.043617	down	hsa-miR-556	0.006278	up
hsa-miR-3926-1	0.001461	down	hsa-miR-7854	0.007002	up
hsa-miR-3926-2	0.002956	down	hsa-miR-1293	0.007069	up
hsa-miR-504	0.001474	down	hsa-miR-4724	0.008888	up
hsa-miR-548aw	0.004953	down	hsa-miR-6510	0.01427	up
hsa-miR-6507	0.038329	down	hsa-miR-7974	0.014417	up
hsa-miR-676	0.010657	down	hsa-miR-449a	0.014478	up
hsa-miR-873	0.026082	down	hsa-miR-1910	0.014907	up
hsa-miR-3934	0.017436	up	hsa-miR-4758	0.0254	up
hsa-miR-6499	0.019667	up	hsa-miR-3194	0.029525	up
hsa-miR-3616	0.021491	up	hsa-miR-940	0.031138	up
hsa-miR-33b	0.021696	up	hsa-miR-579	0.031144	up
hsa-miR-6877	0.023572	up	hsa-miR-7-3	0.032108	up
hsa-miR-4777	0.023592	up	hsa-miR-877	0.033517	up
hsa-miR-7702	0.024974	up	hsa-miR-1181	0.03353	up
hsa-miR-3619	0.025003	up	hsa-miR-937	0.03464	up
hsa-miR-3614	0.038799	up	hsa-miR-3680-1	0.037018	up
hsa-miR-636	0.039294	up	hsa-miR-301b	0.043477	up
hsa-miR-548d-1	0.039563	up	hsa-miR-6793	0.04387	up
hsa-miR-147b	0.039578	up	hsa-miR-6891	0.044216	up
hsa-miR-1537	0.039668	up	hsa-miR-548d-2	0.044259	up
hsa-miR-1224	0.039821	up	hsa-miR-5581	0.044489	up
hsa-miR-3651	0.041106	up	hsa-miR-34b	0.045914	up
hsa-miR-2277	0.041748	up	hsa-miR-1277	0.047288	up
hsa-miR-642a	0.042589	up	hsa-miR-3150b	0.048158	up

Table S5. Correlation Analysis of CDK2 Immune Checkpoint

gene	Immune	r	p	rd
CDK2	CD47	0.486889	8.48E-20	0.4 - 0.6
CDK2	CD276	0.307269	3.52E-08	0.2 - 0.4
CDK2	NCR3LG1	0.277795	7.00E-07	0.2 - 0.4
CDK2	CD80	0.259584	3.76E-06	0.2 - 0.4
CDK2	CD96	0.257152	4.67E-06	0.2 - 0.4
CDK2	TIGIT	0.25476	5.76E-06	0.2 - 0.4
CDK2	CD274	0.23791	2.38E-05	0.2 - 0.4
CDK2	CD86	0.229214	4.76E-05	0.2 - 0.4
CDK2	CTLA4	0.223036	7.66E-05	0.2 - 0.4
CDK2	CD160	0.221909	8.35E-05	0.2 - 0.4
CDK2	CD244	0.181212	0.001379	< 0.2
CDK2	BTLA	0.179513	0.001532	< 0.2
CDK2	ADORA2A	0.171477	0.00249	< 0.2
CDK2	CD28	0.167174	0.003203	< 0.2
CDK2	CD200	0.163847	0.003877	< 0.2
CDK2	CD27	0.158187	0.00532	< 0.2
CDK2	CD48	0.155201	0.006262	< 0.2
CDK2	LAG3	0.143176	0.011749	< 0.2
CDK2	TNFRSF14	0.128078	0.02435	< 0.2
CDK2	TNFRSF18	0.115555	0.042373	< 0.2
CDK2	TMIGD2	0.109405	0.054715	< 0.2
CDK2	CD70	0.08836	0.121149	< 0.2
CDK2	CEACAM1	0.070919	0.213807	< 0.2
CDK2	TNFRSF4	0.033128	0.561826	< 0.2
CDK2	VTCN1	0.027468	0.630531	< 0.2

Table S6. Correlation Analysis of lncRNA Immune Checkpoints

gene	immune	r	p	rd	gene	immune	r	p	rd
CYP4A22-AS1	CD47	0.392031	8.57E-13	0.2 - 0.4	LINC00958	CD47	0.372438	1.33E-11	0.2 - 0.4
CYP4A22-AS1	CEACAM1	0.321042	7.74E-09	0.2 - 0.4	LINC00958	CD70	0.288374	2.49E-07	0.2 - 0.4
CYP4A22-AS1	NCR3LG1	0.252979	6.72E-06	0.2 - 0.4	LINC00958	CD274	0.240507	1.92E-05	0.2 - 0.4
CYP4A22-AS1	TNFRSF14	0.166684	0.003295	< 0.2	LINC00958	CD80	0.225898	6.16E-05	0.2 - 0.4
CYP4A22-AS1	CD244	0.159849	0.004853	< 0.2	LINC00958	CD276	0.210753	0.00019	0.2 - 0.4
CYP4A22-AS1	CD96	0.149494	0.008488	< 0.2	LINC00958	CD86	0.200827	0.000382	0.2 - 0.4
CYP4A22-AS1	CD160	0.145847	0.010255	< 0.2	LINC00958	TIGIT	0.177265	0.001759	< 0.2
CYP4A22-AS1	CD274	0.119677	0.035486	< 0.2	LINC00958	CD96	0.173197	0.002248	< 0.2
CYP4A22-AS1	TMIGD2	0.119487	0.035781	< 0.2	LINC00958	CTLA4	0.170112	0.002699	< 0.2
CYP4A22-AS1	CD200	-0.115943	0.04168	< 0.2	LINC00958	CD244	0.166987	0.003238	< 0.2
CYP4A22-AS1	VTCN1	0.083048	0.145269	< 0.2	LINC00958	TMIGD2	0.131593	0.020674	< 0.2
CYP4A22-AS1	TIGIT	0.078668	0.167774	< 0.2	LINC00958	VTCN1	-0.123103	0.030509	< 0.2
CYP4A22-AS1	CD276	0.077757	0.172768	< 0.2	LINC00958	CD48	0.108298	0.057226	< 0.2
CYP4A22-AS1	LAG3	0.071201	0.211992	< 0.2	LINC00958	NCR3LG1	0.106406	0.061738	< 0.2
CYP4A22-AS1	CD86	0.068479	0.230027	< 0.2	LINC00958	TNFRSF18	0.097201	0.088052	< 0.2
CYP4A22-AS1	CD28	0.066897	0.240999	< 0.2	LINC00958	LAG3	0.093581	0.100602	< 0.2
CYP4A22-AS1	ADORA2A	0.059652	0.2959	< 0.2	LINC00958	CD28	0.063613	0.264932	< 0.2
CYP4A22-AS1	CD27	0.058869	0.302297	< 0.2	LINC00958	CD160	0.050404	0.377242	< 0.2
CYP4A22-AS1	TNFRSF4	0.056486	0.322319	< 0.2	LINC00958	BTLA	0.047938	0.40105	< 0.2
CYP4A22-AS1	BTLA	0.046686	0.413484	< 0.2	LINC00958	TNFRSF14	0.026543	0.642097	< 0.2
CYP4A22-AS1	CD70	0.045374	0.426745	< 0.2	LINC00958	CEACAM1	0.02607	0.648041	< 0.2
CYP4A22-AS1	TNFRSF18	0.044747	0.433167	< 0.2	LINC00958	CD27	0.024337	0.670011	< 0.2
CYP4A22-AS1	CTLA4	0.038291	0.502468	< 0.2	LINC00958	ADORA2A	0.024189	0.671894	< 0.2
CYP4A22-AS1	CD80	0.035116	0.538574	< 0.2	LINC00958	TNFRSF4	0.013301	0.815861	< 0.2
CYP4A22-AS1	CD48	0.013263	0.81638	< 0.2	LINC00958	CD200	0.01047	0.854552	< 0.2

Supplementary Figures

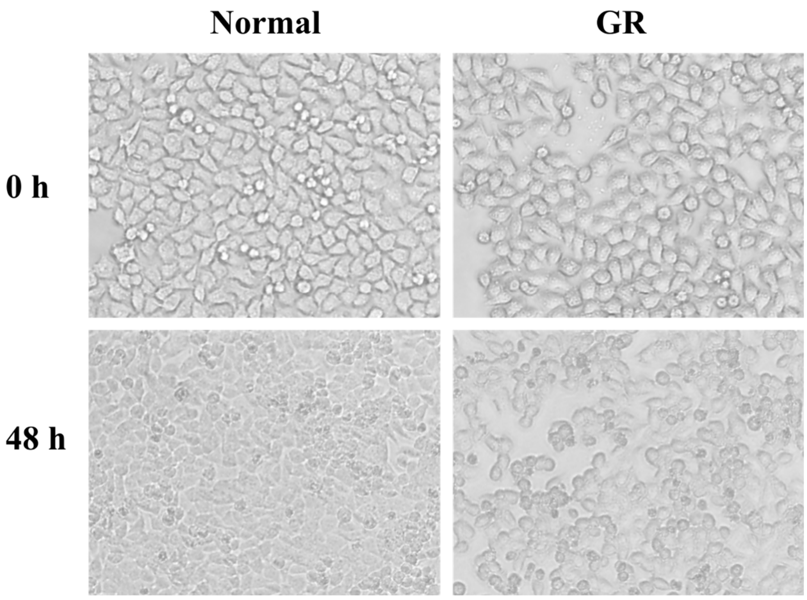

Figure S1. Effects of GR on HeLa cell morphology. Representative images of morphology (bright-field microscopy) after treatment with GR (120 μg/mL) or control for 48 h.

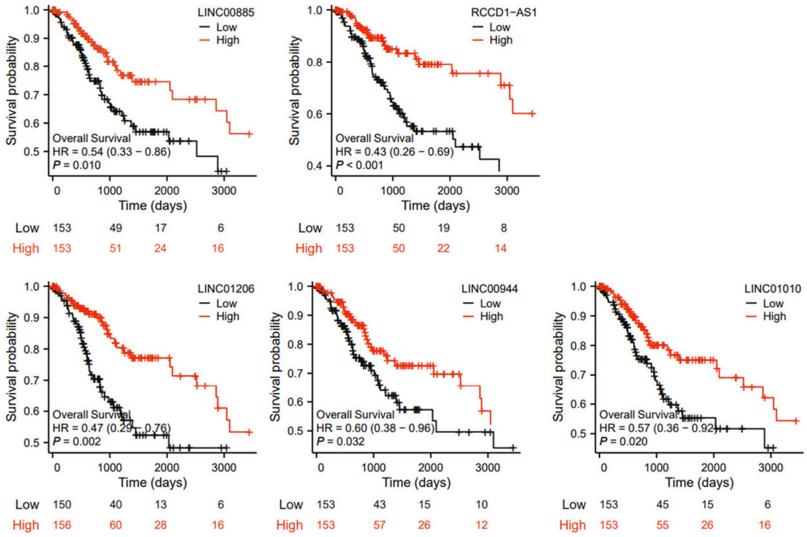

Figure S2. Survival curves of LINC00885, RCCD1-AS1, LINC01206, LINC00944, LINC01010.

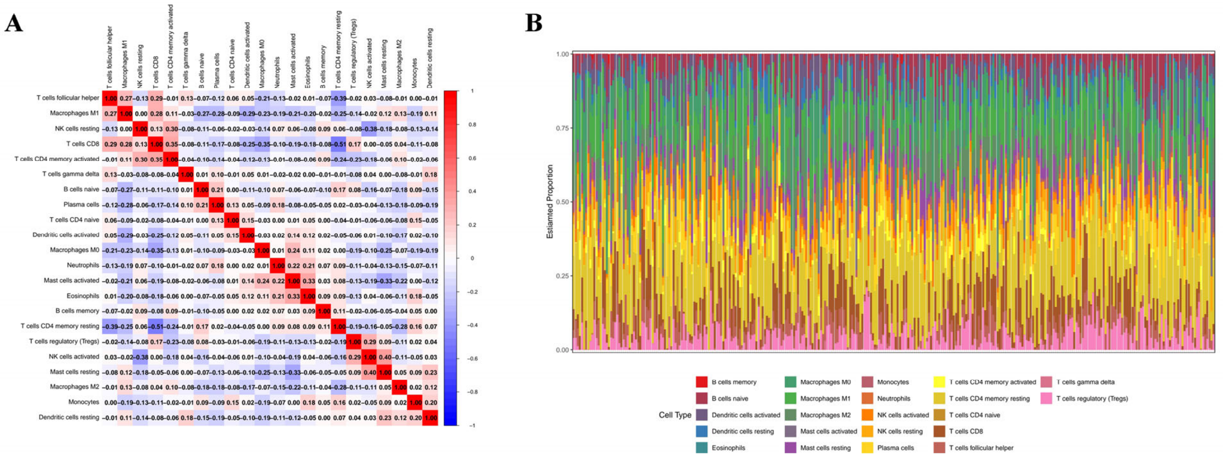

Figure S3. Supplementary data on immune infiltration analysis of TCGA data utilizing the CIBERSORT algorithm. (A) Heatmap of immune cell correlation in the TCGA dataset. (B) Analysis of immune infiltration in TCGA dataset samples.
